# Transgenic mouse model of cutaneous adnexal tumors

**DOI:** 10.1242/dmm.017574

**Published:** 2014-10-10

**Authors:** Yusuke Kito, Chiemi Saigo, Kurabayashi Atsushi, Furihata Mutsuo, Takeuchi Tamotsu

**Affiliations:** 1 Department of Pathology and Translational Research, Gifu University Graduate School of Medicine, Yanagido 1-1, Gifu 501-1194, Japan.; 2 Department of Pathology, Kochi Medical School, Nankoku 783-8505, Japan.

**Keywords:** Cutaneous adnexal tumor, Mouse model, TMEM207

## Abstract

TMEM207 was first characterized as being an important molecule for the invasion activity of gastric signet-ring cell carcinoma cells. In order to unravel the pathological properties of TMEM207, we generated several transgenic mouse lines, designated C57BL/6-Tg (ITF-TMEM207), in which murine TMEM207 was ectopically expressed under a truncated (by ~200 bp) proximal promoter of the murine intestinal trefoil factor (*ITF*) gene (also known as *Tff3*). Unexpectedly, a C57BL/6-Tg (ITF-TMEM207) mouse line exhibited a high incidence of spontaneous intradermal tumors with histopathological features that resembled those of various human cutaneous adnexal tumors. These tumors were found in ~14% female and 13% of male 6- to 12-month-old mice. TMEM207 immunoreactivity was found in hair follicle bulge cells in non-tumorous skin, as well as in cutaneous adnexal tumors of the transgenic mouse. The ITF-TMEM207 construct in this line appeared to be inserted to a major satellite repeat sequence at chromosome 2, in which no definite coding molecule was found. In addition, we also observed cutaneous adnexal tumors in three other C57BL/6-Tg (ITF-TMEM207) transgenic mouse lines. We believe that the C57BL/6-Tg (ITF-TMEM207) mouse might be a useful model to understand human cutaneous adnexal tumors.

## INTRODUCTION

Cutaneous adnexal tumors are a diverse group of neoplasms that exhibit morphological features of differentiation towards a type of adnexal epithelium present in normal skin (for a review, see Sellheyer, 2011).

There has been a notable increase in the incidence rates of cutaneous adnexal tumor since 1980, which is thought to be related to the improved diagnostic strategies and classification of tumors; however, other factors such as UV exposure and immunosuppression might also play a role in the increased incidence (Blake et al., 2010). In fact, immunosuppressed organ transplant recipients have been found to have a greatly increased risk of cutaneous adnexal tumors, especially malignant tumors, compared with apparently immunocompetent recipients (Harwood et al., 2003). However, a pathological approach to experimental research on cutaneous adnexal tumors is required to further elucidate the carcinogenesis of these tumors.

There are many arguments surrounding the classification of adnexal neoplasms (Crowson et al., 2006), which is based on their morphological analogies with normal structures. Histopathological assessment is useful in identifying apocrine differentiation when a tumor cell shows membrane-bound apocrine decapitation secretion. By contrast, it has been often claimed that microscopic examination is insufficient to establish an eccrine lineage. In particular, obtaining a clear diagnosis of malignant adnexal tumors is often difficult owing to their uncharacterized pathological properties (Crowson et al., 2006). In this regard, animal models of cutaneous adnexal tumor are valuable for unraveling the origin of adnexal neoplasms, their pathological features and carcinogenesis, and for developing new treatment strategies, especially for malignant cutaneous adnexal tumors.

TMEM207 was initially identified by a large-scale effort termed the Secreted Protein Discovery Initiative (SPDI), which aimed to find new secreted and transmembrane proteins (Clark et al., 2003). Subsequently, human TMEM207 was found to be overexpressed in many aggressive gastric signet-ring cell carcinomas. An interesting finding is that TMEM207 facilitates tumor invasion, possibly through binding to WWOX, a tumor suppressor molecule, and attenuating the tumor growth suppression activity of the molecule (Takeuchi et al., 2012). Moreover, a mucinous adenocarcinoma of the colon was observed to strongly express TMEM207 (our unpublished observation). Physiologically, TMEM207 expression is relatively restricted to the kidney (Takeuchi et al., 2012). We speculate that ectopically expressed TMEM207 binds to WWOX and is related to gastrointestinal carcinogenesis.

To explore the pathological properties of TMEM207 in gastrointestinal tumors, we originally generated several C57BL/6-Tg (ITF-TMEM207) mouse lines, designated C57BL/6-Tg (ITF-TMEM207), in which murine TMEM207 is ectopically expressed under the proximal promoter (truncated by ~200 bp) of the murine intestinal trefoil factor (*ITF*) gene (also known as *Tff3*). Unexpectedly, a C57BL/6-Tg (ITF-TMEM207) mouse line exhibited a high incidence of spontaneous intradermal tumors with histopathological features that resemble those of various human cutaneous adnexal tumors. In addition, cutaneous adnexal tumors were also found in other three C57BL/6-Tg (ITF-TMEM207) lines. Here, we report this murine model, which might be useful to study the human cutaneous adnexal tumors.

## RESULTS

### Incidence of cutaneous adnexal tumors in a C57BL/6-Tg (ITF-TMEM207) mouse line

Cutaneous adnexal tumors were found in ~14% (14 of 98 female mice) and 13% (13 of 102 male mice) of 6- to 12-month-old heterogenic mice. All the tumors examined in this study were found in the subcutaneous axilla (12 mice), inguinal region (9 mice) or back (6 mice).

**Fig. 1.**
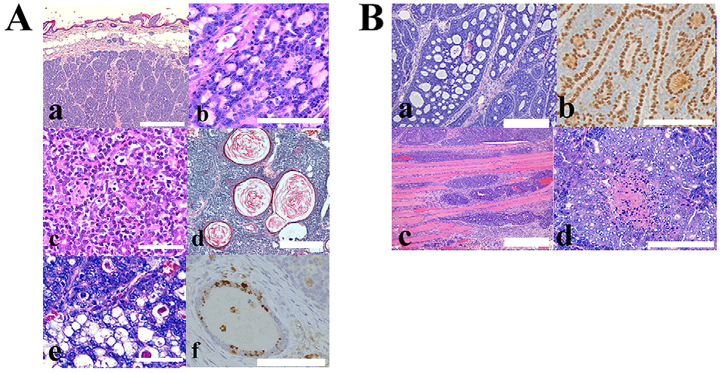
**Representative histopathological findings of the dermal tumors that appeared in the C57BL/6-Tg (ITF-TMEM207) mouse.** (A) Lobules of the basaloid cells arranged in jigsaw or mosaic pattern without connection to the epidermis. These morphological features are characteristic of human cylindromas and spiradenomas (a; scale bar: 1000 μm). (b) Hyaline matrix materials are regularly dispersed in basaloid tumor cells, which is a feature of cylindroma. Scale bar: 50 μm. (c) Trabecular tumor nests composed of small basaloid cells and large cuboidal cells with thin membranous stroma, which is often found in spiradenoma. Scale bar: 100 μm. Follicular (d; scale bar: 500 μm) and sebaceous differentiations (e; scale bar: 100 μm) were partially observed in several tumors. Note the tumor nests with keratin-filled cysts that are found in trichoepithelioma (d). Adipophilin immunoreactivity also indicated sebaceous differentiation (f; scale bar: 50 μm). (B) Cutaneous adnexal tumors with malignant phenotypes were also observed. Cribriform architecture was found in the tumor nest (a). The p63 immunoreactivity was detected in the peripheral tumor cells of the nest, and myoepithelial-like cells faced pseudoglands in the cribriform lesions (b). These features were compatible with those of adenoid cystic carcinomas. Several tumors partially exhibited the invasion to skeletal muscle (c) or comedo-type necrosis (d), which is known to be malignant characteristics of cutaneous adnexal tumors. (Ba–d) Scale bars: 100 μm.

The representative histopathological features of the tumors are shown in Fig. 1. The morphological appearances of the tumors greatly varied; however, almost all the tumors examined exhibited, at least partially, differentiation into sweat gland units. Moreover, combined morphological features with human cylindroma and spiradenoma were often observed (Fig. 1Aa–c). In these tumors, basaloid cells grew with so-called puzzle-like and/or micronodular arrays. Several of the tumors also resembled adenoid cystic carcinoma with tubular and cribriform patterns (Fig. 1Ba). Immunoreactivity using a specific antibody against p63 was observed in the abluminal myoepithelial-like cells but not in the luminal cells (Fig. 1Bb), which is a feature of adenoid cystic carcinomas, as reported previously (Emanuel et al., 2005). In addition, some tumors partially exhibited follicular (Fig. 1Ad) and sebaceous differentiations (Fig. 1Ae). Adipophilin immunoreactivity also indicated the sebaceous differentiation of several tumors (Fig. 1Af) (Ostler et al., 2010). A poorly differentiated carcinoma that markedly invaded surrounding tissues was also found in the focal cutaneous adnexal tumor area (Fig. 1Bc,Bd). However, no tumor metastasis was found.

**TRANSLATIONAL IMPACT**

**Clinical issue**

Cutaneous adnexal tumors are a diverse group of neoplasms that exhibit morphological features of different types of skin appendages (adnexa), such as hairs, follicles and sebaceous glands. However, the question that remains to be unraveled is the exact origin of an adnexal tumor. There are also many arguments surrounding the classification of cutaneous adnexal tumors based on their morphological analogies with normal structures of the skin. There has been a notable increase in cutaneous adnexal tumor incidence rates, which is thought to be related to the improved diagnostic strategies and classification of these tumors. However, diagnosing malignant versus non-malignant adnexal tumors is often difficult to obtain owing to the uncharacterized pathological properties of these tumors. In this regard, animal models of cutaneous adnexal tumors are valuable for unraveling the origin, the pathological features and the carcinogenesis of these tumors, as well as for developing new treatments, especially for the malignant forms.

**Results**

C57BL/6-Tg (ITF-TMEM207) transgenic mouse lines were generated, in which the TMEM207 gene (which is overexpressed in several human carcinomas) was ectopically expressed under a truncated promoter from the murine intestinal trefoil factor (*ITF*) gene. Three of these lines exhibited malignant invasive cutaneous adnexal tumors, and one in particular exhibited a high incidence of spontaneous intradermal tumors with histopathological features that resembled those of various human cutaneous adnexal tumors, including those found in Brooke–Spiegler syndrome (BSS, a genetic condition associated with predisposition to cutaneous adnexal tumors). It is suggested that the ectopic expression of TMEM207 in sebaceous gland cells and/or hair follicle bulge cells might be directly associated with cutaneous adnexal carcinogenesis in the present transgenic mice.

**Implications and future directions**

The outcome of the present study provides three important findings. First, C57BL/6-Tg (ITF-TMEM207) mice might be a novel model to understand the pathophysiology of human skin appendage tumors, including those with a malignant invasion phenotype. Second, hair follicle bulge cells, which are multipotent stem cells that support hair follicle cycling and repopulation, might be the cells that give rise to various skin appendage tumors, and the truncated *ITF* promoter used in this study might be useful to express other cancer-associated exogenous genes specifically in hair follicle bulge stem cells, and further investigate their role in adnexal tumor development. Third, because the phenotype of the C57BL/6-Tg (ITF-TMEM207) mouse appears to be similar to that of human BSS, it would be interesting to examine whether mutations in the *TMEM207* gene are also present in some BSS cases.

Although the benign adnexal tumors in the mice exhibited a combination of histopathological features consistent with various human adnexal tumors, we believe that all of the 12 axilla tumors, four of the nine inguinal tumors and four of the six back tumors mainly exhibited the cylindroma or spiradenoma phenotypes. Among these tumors, two of the axilla and three of inguinal tumors also exhibited a partial trichoepithelioma morphology. We found benign sebaceous tumors in one of the nine inguinal and two of the six back tumors. We found malignant adnexal tumors with adenoid cystic patterns, invasion and/or comedonecrosis in four of the inguinal tumors. Interestingly, all of the malignant tumors were *de novo* tumors without any benign adnexal tumor components.

Based on these histological features, we conclude that the C57BL/6-Tg (ITF-TMEM207) mouse line exhibited a high incidence of cutaneous adnexal tumors, which sometimes exhibit the malignant invasive phenotype.

### Exogenous TMEM207 expression in skin of a C57BL/6-Tg (ITF-TMEM207) mouse line

Although *ITF* is selectively expressed in intestinal goblet cells, the proximal promoter of the *ITF* gene used in this study (Itoh et al., 1999) is insufficient to recapitulate the exquisite tissue- and cell-specific expression of the native *ITF* promoter but rather allows gene expression in various tissues including the gastrointestinal tract, as it lacks a goblet cell silencer inhibitor element (Iwakiri and Podolsky, 2001). Therefore, we further investigated whether TMEM207 was expressed in the non-tumorous skin and adnexal tumors of the C57BL/6-Tg (ITF-TMEM207) mouse and its wild-type littermate. Interestingly, immunohistochemical staining using rabbit antibody specific to TMEM207 demonstrated expression of TMEM207 in non-tumorous skin tissues of the C57BL/6-Tg (ITF-TMEM207) mouse.

**Fig. 2.**
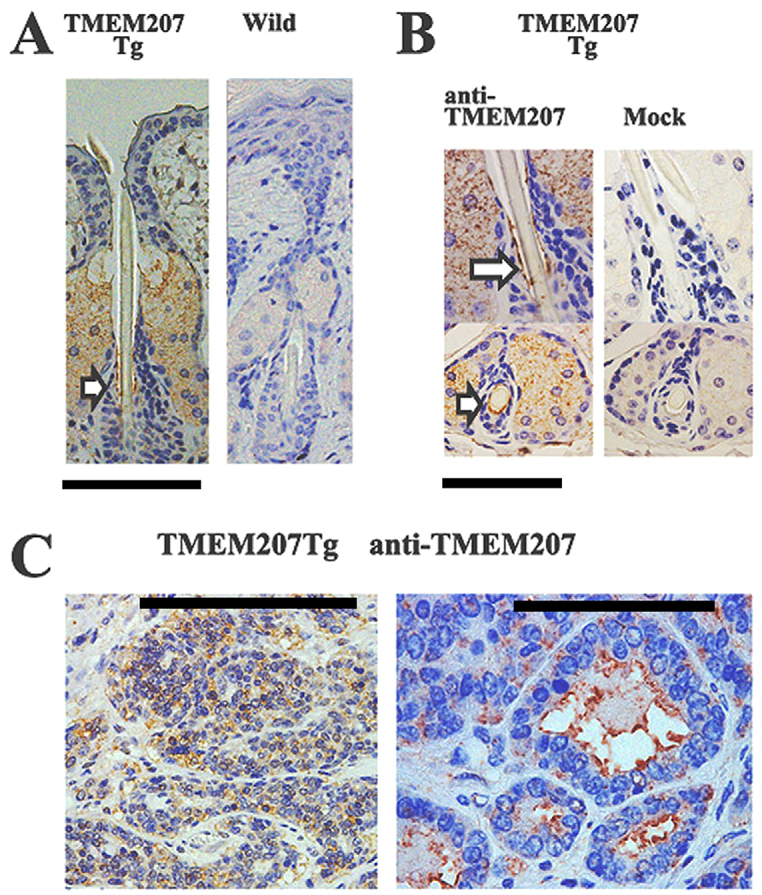
**Immunohistochemical staining using an antibody specific to TMEM207.** (A,B) The TMEM207 immunoreactivity was observed in the sebaceous gland cells and hair follicle bulge cells of the C57BL/6-Tg (ITF-TMEM207) mouse (white arrows indicates the bulge region). In contrast, no substantial immunoreactivity was found in the skin of a wild-type littermate (A, Wild) or using the control antibody (B, Mock). (C) Dermal tumor of the C57BL/6-Tg (ITF-TMEM207) mouse was stained with antibody specific to TMEM207. Scale bars: 100 μm (A; C, left), 50 μm (B), 10 μm (C, right).

As depicted in Fig. 2A,B, TMEM207 immunoreactivity was not only found in sebaceous gland cells but also in hair follicle bulge cells, which are the repository of multipotent stem cells that support hair follicle cycling and repopulate the interfollicular epidermis and sebaceous epithelium (Cotsarelis et al., 1990). Given that we did not find any substantial TMEM207 immunoreactivity in the wild-type mouse skin tissues, TMEM207, which was found in sebaceous gland cells and hair follicle bulge cells of the transgenic mouse, might be exogenously expressed under the short proximal promoter of *ITF* used in this study. As expected, TMEM207 was expressed in cutaneous adnexal tumor cells (Fig. 2C).

### Chromosome location of the ITF-TMEM207 construct

A transgene often confers a gain of function, but a loss of function can occur if the integrated transgene interrupts another gene. We therefore performed ‘GeneWalking’ to identify the chromosomal region in which the ITF-TMEM207 construct was inserted, using the Universal GenomeWalker Kit (Clontech-Takara, Ohtsu, Japan). The ITF-TMEM207 construct appeared to be inserted to a major satellite repeat sequence at chromosome 2, in which no definite coding gene was found. An independent experiment using APAgene™ GOLD-RT Genome Walking kits (Bio S&T, Montreal, Quebec, Canada), which do not require the restriction enzyme treatment, gave an identical result.

Taken together with the finding that cutaneous adnexal tumors were also found in three other C57BL/6-Tg (ITF-TMEM207) mouse lines, we think that enforced TMEM207 expression is directly associated with cutaneous adnexal carcinogenesis in the transgenic mice presented in this study.

## DISCUSSION

In this study, we described a novel transgenic mouse line, C57BL/6-Tg (ITF-TMEM207), in which cutaneous appendage tumors form spontaneously at high incidence. Notably, the pathological features of these tumors are very similar to those of human skin appendage tumors. All tumors formed intradermal masses that usually resembled human cylindroma or spiradenoma tumors, and which were histopathologically characterized as mosaic-like tumor nests of basaloid cells. Several of the tumors resembled human adenoid cystic carcinoma with tubular and cribriform patterns. In addition, some tumors partially exhibited apocrine, follicular and sebaceous differentiations. Notably, human skin appendage tumors also often exhibit overlapping features with follicular and sebaceous gland differentiation. Therefore, we believe that this mouse will be a very suitable model for the pathological properties of human appendage tumors.

In the cutaneous adnexal tumors, we found adenoid cystic carcinoma-like malignant tumors (Fig. 1Ba,b) and malignant tumors with features of salivary-duct type adenocarcinoma (Fig. 1Bc). We think that advances in research and the accumulation of cases, including those from animal models, means that cutaneous salivary-gland type adenocarcinoma will be included in the category of cutaneous adnexal tumors, similar to mammary gland and lung-bronchial tumors. Notably, the intradermal tumor in the transgenic mouse described in the present study demonstrated only a few morphological features of eccrine glands. In mice, the footpad is the only anatomic region that contains eccrine glands. In contrast, humans have eccrine glands over most of the surfaces of the body. This evolutionary difference might be responsible for the limited eccrine gland differentiation exhibited by the tumors.

A gene walking assay showed that the plasmid construct was not inserted into any definite coding region in the C57BL/6-Tg (ITF-TMEM207) mouse. We also found cutaneous appendage tumors in another three C57BL/6-Tg (ITF-TMEM207) transgenic mouse lines. Taken together, exogenous expression of TMEM207 in sebaceous gland cells and/or hair follicle bulge cells might be responsible for the formation of the cutaneous appendage tumors observed in this study. Notably, we also found TMEM207 immunoreactivity in human skin appendage tumors, including cylindroma and spiradenoma (data not shown), as well as in the intradermal tumors of C57BL/6-Tg (ITF-TMEM207) mice.

Hair follicle bulge cells are well characterized as a repository of multipotent stem cells that support hair follicle cycling and repopulate the interfollicular epidermis and sebaceous epithelium (Cotsarelis et al., 1990). It is likely that TMEM207 expression in hair follicle bulge cells is important for development of cutaneous appendage tumors. Therefore, we postulate that the origin cells of adnexal tumors, which has long been an unresolved issue in dermatopathology, might be hair follicle bulge cells.

The present study also reveals a new promoter activity of the truncated *ITF* sequence. Initially, we expected that ubiquitous enforced expression of TMEM207 would be observed in the gastrointestinal tract by using this truncated ITF promoter because it lacks a goblet cell silencer inhibitor element. As demonstrated in Fig. 2, this truncated ITF sequence induced exogenous TMEM207 expression in hair follicle bulge cells and sebaceous gland cells. We observed exogenous TMEM207 expression in the hair follicle bulge cells of another three C57BL/6-Tg (ITF-TMEM207) transgenic mouse lines (data not shown). Therefore, the truncated *ITF* promoter used in this study might be useful to express exogenous proteins and study the biological role of hair follicle bulge cells.

Brooke–Spiegler syndrome (BSS, OMIM number 605041) is an autosomal dominant predisposition to cutaneous adnexal tumors (Brooke, 1892; Spiegler, 1899), that is, tumors with combined features of cylindroma and spiradenoma, sometimes with focal trichoepithelioma components (Kazakov et al., 2005). Although most cases of BSS harbor a mutation in the cylindromatosis (*CYLD*) gene (Bignell et al., 2000; Zhang et al., 2004; Sima et al., 2010), some cases do not (Ponti et al., 2012). Because the phenotype of the C57BL/6-Tg (ITF-TMEM207) mouse appears to be similar to that of human BSS, it would be interesting to examine mutations of the *TMEM207* gene in BSS negative for mutations in the *CYLD* gene. Furthermore, the potential for a relationship between TMEM207 and CYLD proteins needs to be further examined.

In conclusion, the present study provides three important findings. First, C57BL/6-Tg (ITF-TMEM207) mice might be a novel model for human skin appendage tumors, including those with a malignant invasion phenotype. Second, hair follicle bulge cells might be the originating cells of various skin appendage tumors. Third, the truncated *ITF* promoter might be useful for expressing exogenous genes in hair follicle bulge stem cells. In addition, our results suggest a possible relationship between TMEM207, CYLD and BSS.

## MATERIALS AND METHODS

### Generation of the C57BL/6-Tg (ITF-TMEM207) mouse line

The detailed procedure for generation of the transgenic mouse with a C57BL/6 background and cassette vector has been reported previously (Takeuchi et al., 1997). To generate the C57BL/6-Tg (ITF-TMEM207) mouse, we prepared a plasmid containing the full coding region of the murine *Tmem207* gene followed by the proximal promoter of the murine *ITF* gene. Briefly, a truncated 219-bp promoter region of murine *ITF* was obtained by PCR from C57BL/6 mouse genomic DNA by using the following primers: sense 5′-AGTCTGCTTCTAGACTAGGTGTACAC-3′, and antisense 5′-GCCCTTTTATAGCCATGTGTTTGCTGGG-3′. First, the truncated promoter region was subcloned into a previously reported cassette vector (Takeuchi et al., 1997) harboring a poly(A) signal, which was verified by sequencing. Subsequently, the entire coding region of murine *Tmem207* cDNA was amplified by RT-PCR from C57BL/6 kidney tissue RNA and inserted beneath the *ITF* promoter. Synthesis of first-strand cDNA was primed with random hexamers by using an RNA Long and Accurate PCR kit (TaKaRa, Ohtsu, Japan). Primers used to obtain *Tmem207* cDNA were: sense 5′-ATCTGGGTACATCTTTCTTTTTAG-3′, and antisense 5′-CTGCTGCATCTGGATAAAAT-3′. After verifying the entire ITF-TMEM207 gene sequence, the construct was cut out with *Bgl*II and *SphI* restriction enzymes, and the construct fragment was microinjected into pronuclei of fertilized C57BL/6 oocytes by using standard procedures. The resulting founder transgenic mouse was transferred to a specific pathogen-free housing.

Tail biopsies were digested with proteinase K (TaKaRa) and genotyping was performed by PCR to screen for transgenic founder animals as well as routine genotyping. The PCR primers used for tail genotyping were: 5′-AGTCTGCTTCTAGACTAGGTGTACAC-3′ and 5′-ATACCAGCCATCAGGATATCGCTCGTC-3′.

The experimental protocol was approved by the Animal Care Committee of Kochi Medical School, Kochi, Japan and Gifu University Graduate School of Medicine, Gifu City, Japan.

### Immunohistochemical staining

The detailed procedure for immunohistochemical staining, including the preparation (Takeuchi et al., 2000) and characterization of the rabbit antibody specific to TMEM207, which can detect both the human and murine TMEM207 proteins, has been described previously (Takeuchi et al., 2012). Antibodies specific to p63 and adipophilin were purchased from Dako (no. M7274, Kyoto, Japan) and American Research Products (no. 03-610102, Waltham, MA), respectively. Tissues were immunostained with antibodies using the ImmPRESS™ polymerized reporter enzyme staining system (Vector Laboratories, Burlingame, CA), as previously reported (Bai et al., 2014).

### Gene walking

To identify the chromosomal insertion region of the ITF-TMEM207 construct, we employed the Universal GenomeWalker Kit (TaKaRa) and the APAgene™ GOLD-RT Genome Walking Kit (Bio S&T, Montreal, Quebec, Canada). The procedures were performed according to the manufacturer’s instructions.
